# Near-Cognate Codons Contribute Complexity to Translation Regulation

**DOI:** 10.1128/mBio.01820-17

**Published:** 2017-11-07

**Authors:** N. Louise Glass

**Affiliations:** a Plant and Microbial Biology Department, University of California, Berkeley, California, USA; b Lawrence Berkeley National Laboratory, Berkeley, California, USA

**Keywords:** cross-pathway control, near-cognate codons, translation, uORFs

## Abstract

The interplay between translation initiation, modification of translation initiation factors, and selection of start sites on mRNA for protein synthesis can play a regulatory role in the cellular response to stress, development, and cell fate in eukaryotic species by shaping the proteome. As shown by Ivanov et al. (mBio 8:e00844-17, 2017, https://doi.org/10.1128/mBio.00844-17), in the filamentous fungus *Neurospora crassa*, both upstream open reading frames (uORFs) and near-cognate start codons negatively or positively regulate the translation of the transcription factor CPC1 and production of CPC1 isoforms, which mediate the cellular response to amino acid starvation. Dissecting the physiological roles that differentiate cellular choice of translation initiation is an important parameter to understanding mechanisms that determine cell fate via gene regulation and protein synthesis.

## COMMENTARY

Translational control of protein synthesis in eukaryotic cells is a well-known phenomenon, with noncanonical initiation events controlling gene expression of coding regions. In eukaryotic species, translation initiation is mediated by the assembly of a ribosomal preinitiation complex (43S preinitiation complex [PIC]) followed by scanning of the 5′ untranslated region (UTR) of mRNAs for a start codon (AUG) (1, 2). At an AUG start codon, the 80S initiation complex forms by modification and binding of the large (60S) ribosomal subunit to a modified PIC, resulting in the initiation of protein synthesis. Although AUG start codons begin the main coding sequence of many mRNAs, other mRNAs contain upstream open reading frames (uORFs) in the 5′ leader region that have been shown to confer regulatory properties (2–4). Because reinitiation of translation is generally inefficient in eukaryotes and ribosome stalling can occur at some uORFs, the translation of a uORF generally results in attenuation of translation of a downstream protein coding ORF, although both positive and negative effects on reinitiation can occur. Thus, the choice of a start codon by a scanning ribosome can play a major role in shaping the cellular proteome by affecting translational efficiency of coding mRNAs (5–7). In mammalian cells, genome-wide sequencing of 5′ UTRs revealed that uORFs are very frequent (~40% of mammalian mRNAs) (8), suggesting regulatory effects of these uORFs. Importantly, an association with some human genetic diseases has been found for mutations within the uORFs that positively or negatively affect translation of protein coding ORFs (9).

Despite the widespread occurrence of uORFs in eukaryotic mRNA, biochemical and genetic evidence for a regulatory role for 5′ leader region uORFs on translation of downstream ORFs has been shown for relatively few genes. One of these genes is *GCN4* of *Saccharomyces cerevisiae*, which encodes a transcription factor important for modulating the cellular response to amino acid starvation (10). Two uORFs in the 5′ UTR of *GCN4* mRNA play pivotal regulatory roles. uORF1 acts as a positive regulatory element to facilitate reinitiation at the start codon for the *GCN4* protein coding ORF, while uORF4 strongly inhibits the translation of *GCN4* by preventing reinitiation. Homologs of *GCN4* in other fungi contain at least two uORFs believed to have similar regulatory functions as the *S. cerevisiae GCN4* uORFs. In addition to these regulatory uORFs, kinases such as Gcn2 also play a regulatory role in *GCN4* expression; phosphorylation of the translation initiation factor eIF2α (α subunit of eukaryotic initiation factor 2) by Gcn2 kinase increases reinitiation at the *GCN4* protein coding start codon in response to amino acid limitation (11).

**FIG 1:**
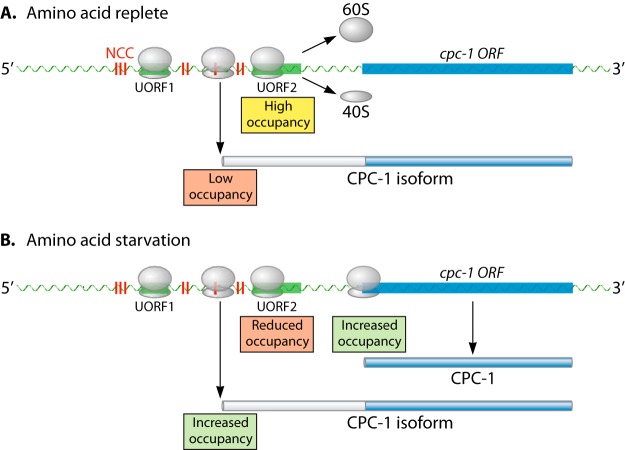
Translation initiating from NCCs in the ~700-nucleotide 5′ leader region of *cpc-1* results in CPC1 isoforms. (A) Translation of *cpc-1* mRNA under amino acid-replete conditions is regulated by upstream ORFs (uORFs). Ribosome occupancy of uORFs (particularly of uORF2) strongly reduces translation initiation at the AUG codon of the *cpc-1* ORF. However, a low level of translation initiation can occur from near-cognate codons (NCCs) in the 5′ leader region that are in frame with the *cpc-1* ORF and result in the formation of CPC1 isoforms. (B) Under amino acid starvation conditions, ribosome occupancy of uORF2 is reduced and translation initiation is increased for the *cpc-1* ORF, resulting in production of CPC1. Additionally, an increase in ribosome occupancy at upstream NCCs is observed, resulting in a potential increase in CPC1 isoforms.

The *mBio* article by Ivanov et al. (12) provides elegantly designed experimental evidence that supports the regulatory role of uORFs of the *GCN4* homolog in the filamentous fungus *Neurospora crassa*, the so-called cross-pathway control (*cpc*-*1*) gene (13). As shown by biochemical experiments, including the *in vitro* mapping of ribosomes on mRNA for *cpc-1*, the function of uORFs in regulating the initiation of translation at the *cpc-1* start codon is consistent with the positive and negative regulatory functions of uORFs in *S. cerevisiae GCN4* (Fig. 1). This result confirms the evolutionary conservation of uORFs in translational regulation in response to amino acid starvation among fungi.

The presence and regulatory consequences of the uORFs in the 5′ leader region of *GCN4/cpc-1* are conserved between *S. cerevisiae* and *N. crassa*. However, the 5′ *cpc-1* leader region in *N. crassa* lacks any in-frame stop codons, a feature that is missing from the 5′ UTR of *S. cerevisiae GCN4*. A long 5′ leader sequence that lacks coding region in-frame stop codons is also conserved among 100 *cpc-1* homologs in the genomes of filamentous ascomycete fungi (Pezizomycotina) and some Basidiomycota species. Importantly, in these 5′ leader sequences, multiple non-AUG near-cognate codons (NCCs) occur (Fig. 1), which differ from an AUG start codon by a single base. NCCs can initiate translation, albeit at lower frequencies than AUG start codons, owing to destabilization of the PIC at NCCs. Additionally, the NCCs in 5′ leader sequences of *cpc-1* homologs in filamentous fungi are in frame with the start codon for *cpc-1*. Translation initiation at these NCCs would result in longer isoforms of the CPC1 protein. Importantly, the predicted N-terminal extension of CPC1 among homologs in Pezizomycotina species shows some amino acid conservation. Indeed, the codons in these 5′ leader regions are subject to purifying selection, indicating a functional role for these newly discovered CPC1 isoforms.

Using elegant *in vitro* translation assays and ribosome profiling (14), Ivanov et al. (12) showed that a number of these NCCs in the 5′ leader region of *cpc-1* are able to initiate translation. Additionally, a fusion between the *cpc-1* 5′ leader containing the NCC and the ORF for luciferase showed a higher-than-predicted molecular weight for luciferase, consistent with translation initiating at NCCs to produce longer protein isoforms. Importantly, Ivanov et al. (12) showed that *in vitro* translation from four of these NCCs bypassed the inhibitory effect of the uORF2 on *cpc-1* translation (which impacts initiation at the downstream CPC1 AUG start codon). Ribosome profiling experiments showed that both uORF1 and uORF2 were heavily translated but that additional ribosome footprints in the 5′ region of the *cpc-1* were predominantly in the CPC1 coding frame, indicating that translation initiation occurred at the upstream NCCs as well as the downstream CPC1 AUG start codon *in vivo*.

To determine if amino acid starvation affected use of NCCs as translation initiation sites and thus production of CPC1 isoforms, Ivanov et al. (12) performed ribosome profiling after treating *N. crassa* cells with 3-aminotriazole (3-AT), which induces starvation for histidine. In treated cells, ribosomes were relatively more abundant in the coding region of *cpc*-*1* and relatively less abundant in the inhibitory uORF (uORF2). Importantly, an increase in ribosome density was observed in regions associated with NCCs (Fig. 1), consistent with production of longer CPC1 isoforms under amino acid starvation conditions. These data indicate that, in addition to translational control of *cpc-1* by uORFs and regulatory roles of conserved Gcn2 kinases, filamentous fungi also display translational mechanisms that produce different CPC1 isoforms not subject to translation inhibition by uORFs. Thus, noncanonical initiation of translation at NCCs can confer regulatory properties that have the potential to result, in the case of *cpc-1*, in a significant shift in the physiology of cells due to the increase in CPC1 abundance and transcriptional activity.

The presence of NCCs in the 5′ leader sequences of a number of regulatory genes has been identified in a variety of eukaryotic species (4, 6, 15, 16). Understanding the physiological conditions that control initiation at NCCs has broad implications for gene regulation and protein synthesis. The use of alternative translation initiation sites in response to stress, development, cell fate, or other regulatory processes can provide an expansion and remodeling of the cellular proteome, revealing hidden alternative coding potential. The conserved nature of the coding sequences of the CPC1 isoforms that result as a consequence of translation initiation at NCCs and the genetic, biochemical, and genomic tools available for *N. crassa* (17) make this an excellent model system by which to decipher principles of translation initiation at NCCs and the interplay between regulatory uORFs and modification of translation initiation factors that shape the cellular proteome.
